# Spotted fever group rickettsiae in ticks of migratory birds in Romania

**DOI:** 10.1186/s13071-016-1565-7

**Published:** 2016-05-20

**Authors:** Ioan-Daniel Mărcuţan, Zsuzsa Kalmár, Angela Monica Ionică, Gianluca D’Amico, Andrei Daniel Mihalca, Cozma Vasile, Attila D. Sándor

**Affiliations:** Department of Parasitology and Parasitic Diseases, Faculty of Veterinary Medicine, University of Agricultural Sciences and Veterinary Medicine, Calea Mănăştur 3-5, Cluj Napoca, Romania

**Keywords:** Migratory birds, Ticks, Rickettsiae, Eastern Europe

## Abstract

**Background:**

Birds are important hosts and dispersers of parasitic arthropods and vector-borne zoonotic pathogens. Particularly migratory species may carry these parasites over long distances in short time periods. Migratory hotspots present ideal conditions to get a snapshot of parasite and pathogen diversity of birds migrating between continents. The aim of this study was to investigate the presence and diversity of *Rickettsia* spp. in ticks collected from birds at a migratory hot-spot in the Danube Delta, Romania, eastern Europe.

**Methods:**

DNA was extracted from ticks that were collected from migratory birds in the Danube Delta during migratory seasons in 2011–2012. Two 360 bp  fragments of the 16S ribosomal RNA gene and a 381 bp  fragment Gene gltA were PCR amplified and analyzed by sequence analysis (performed at Macrogen Europe, Amsterdam, The Netherlands). Nucleotide sequences were compared to reference sequences available in the GenBank database, using Basic Local Alignment Search Tool.

**Results:**

Four hundred ticks of four different species were found on 11 bird species. The prevalence of *Rickettsia* spp. infection was 14 % (56/400, CI: 11.7–29.1), with significantly more nymphs hosting rickettsial infection compared to larvae (48 *vs* 7; *P* < 0.001). Significantly more ticks in nymphal stage were hosting *Rickettsia* spp. infection in spring, than in autumn. Four different genospecies were found: *R. monacensis* (29 ticks), *R. helvetica* (13), *R. massiliae* (3) and *R. slovaca* (2). The seasonal distribution of different *Rickettsia* spp. was heterogeneous; with most of the *R. monacensis-*infected ticks were found in spring, while more *R. helvetica* were found in autumn than spring. *R. massiliae* was found only in autumn and *R. slovaca* was found only in spring.

**Conclusion:**

This study has shown that birds migrating through eastern Europe may carry ticks infected with a high diversity of rickettsial pathogens, with four *Rickettsia* spp. recorded. Migratory direction was important for pathogen burden, with seasonal differences in the occurrence of individual *Rickettsia* species. Here we report the first individual records of different *Rickettsia* spp. in *H. concinna* (*R. monacensis*)*, I. arboricola* (*R. helvetica, R. massiliae*) and *I. redikorzevi* (*R. helvetica*) and also the first geographical record of occurrence of R. *massiliae* in Romania, representing the easternmost observation on the continent.

## Background

Migratory birds can act as long distance carriers of vectors and pathogens of zoonotic potential [1] and may facilitate their transmission to humans, wildlife and domestic animals through their mobility, potentially exerting a high influence on the geographic distribution of pathogens and vectors [2]. Wild birds are hosts for various ectoparasites including ticks, which are competent vectors for a number of pathogens causing diseases in humans and animals [3]. Birds are known to play an important role in distributing ticks and their associated pathogens within and between continents [4] but they can also act as reservoir hosts in natural foci of disease [5]. In Europe, migratory birds are commonly hosting a number of tick species, with species of the genera  *Ixodes, Hyalomma*, *Rhipicephalus* and *Haemaphysalis* most frequently collected [6–8]. Ixodid ticks having a wider host spectrum are important vectors for several agents of human and animal pathogens, including *Borrelia* spp., *Anaplasma* spp., *Rickettsia* spp., *Francisella tularensis* and *Babesia* spp. [1]. In Romania, wild birds were found to be parasitized by several tick species, i.e. *Ixodes ricinus, I. arboricola, I. redikorzevi, Haemaphysalis concinna, Hyalomma marginatum* and *Rhipicephalus sanguineus* [9, 10].

The distribution and frequency of occurrence of certain *Rickettsia* spp. in ticks is mostly well known in the southern and western part of Europe. However, current knowledge on the diversity of *Rickettsia* spp. in eastern Europe is almost absent [11]. There are only two studies in Romania detailing the occurrence of *Rickettsia* spp. group, with [12] demonstrating the presence of *R. conorii* in humans, based on serology, and [13] presenting details on four *Rickettsia* species in questing and engorged ticks collected from mammalian hosts.

There are two major bird migratory routes in eastern Europe, connecting the north-eastern part of the continent, as well western Siberia to the Mediterranean region and these two join into the Eastern Mediterranean Flyway on the territory of Romania (in the Danube Delta). This region is one of the most important migratory stop-over sites in Europe, with more than 300 species of migratory birds being recorded in most years. Not only the diversity, but also the numbers passing and stopping-over is high, with an estimated two million birds using the diverse habitats of the region twice a year [14]. In addition, 15 tick species were recorded in this area [9, 15]. Thus, the region provides a unique setting to assess the importance of birds in the cycling of Spotted Fever Group *Rickettsia* (SFG *Rickettsia*). There are no data on rickettsial infections of birds or their ticks from Romania. The aim of this study was to investigate the presence of *Rickettsia* spp. in ticks collected from birds at a migratory hot-spot in the Danube Delta.

## Methods

The ticks assessed in this study were collected from the Danube Delta, south-east Romania. Here, passerines were captured during the migratory seasons (spring and autumn), in the years 2011 and 2012. The birds were captured with the use of ornithological mistnets (five shelf type, 12 m long, Ecotone Inc., Poland) erected in different habitats around the ornithological field laboratory, located at Grindul Lupilor, the Danube Delta Biosphere Reserve, Constanța county, Romania (44.695848N, 28.939243E). The fauna of the area is rich in breeding water birds and passerines breeding in reed-beds, with high numbers of migrant passerines using the area as short-time stop-over location in both migratory seasons [10]. The trapping lasted for one week in each season and occurred in April and October in both years, targeting the migration peak of small to medium sized passerines in the region (for further details see [10]). Ticks were collected from the head and body of birds with fine tweezers and preserved in 96 % ethanol using a separate vial for each bird. Ticks were identified using standard morphological keys [16, 17] under a stereomicroscope.

For studying the seasonal distribution of ticks and the hosted pathogens, the two trapping seasons in any given year (spring and autumn) were considered separately. The bird species were grouped according to their status in the region: migrants (occurring for short periods lasting from a few days to a few weeks in spring and/or autumn) and breeding birds (either migratory or resident, spending the boreal summer in the region).

### DNA extraction and PCR

DNA extraction was performed using a commercial DNA extraction kit (DNAEasyBlood & Tissue Kit, QIAGEN, Hilden, Germany) according to the manufacturer’s recommendations. The quantity and purity of DNA were assessed using spectrophotometer analyses (NanoDropTechnologies model ND-1000 Inc., Wilmington, DE, USA). Briefly, each tick was submitted to DNA extraction and to polymerase chain reaction (PCR) using 10 pmol/μl from each primer (forward: 5′-AAC GCT ATC GGT ATG CTT AAC A-3′, reverse: 5′-ACT CAC TCG GTA TTG CTG GA-3′) to amplify a 360 bp fragment of the 16S ribosomal RNA gene using 2× Green Master Mix (RovalabGmBH, Teltow, Germany) and for Gene gltA, 381 bp fragment (Primers Rsfg877: 5′-GGG GGC CTG CTC ACG GCG G-3′ and Rsfg1258: 5′-ATT GCA AAA AGT ACA GTG AAC A-3′). The PCR reaction was performed according to a previously described protocol [18]. For quality control of the reactions, positive and negative controls were included: for positive control we used *Rickettsia* DNA previously confirmed by sequencing, while negative control was assured using tap-water instead of DNA. In addition amplicons were visualized by electrophoresis in 1.5 % agarose gel stained with SYBR ®Safe DNA gel stain (Invitrogen, Carlsbad, CA, USA).

### DNA sequencing

PCR products were purified from gel using QIAquick PCR purification kit (QIAGEN, Hilden, Germany) and analyzed by sequence analysis (performed at Macrogen Europe, Amsterdam, The Netherlands). Nucleotide sequences were compared to reference sequences available in the GenBank^TM^, using Basic Local Alignment Search Tool (BLAST) analysis.

### Statistical analysis

Exact confidence intervals (CIs) for the prevalence rates at the 95 % level were calculated using the software Quantitative Parasitology 3.0 [19]. Sample prevalence data were analyzed using Fisher’s exact test. Differences were considered significant when *P* < 0.05.

### Ethical approval

The study was carried out according to the national wildlife welfare regulations (OUG57/2007). Licence for bird ringing was provided by the Romanian Ornithological Centre (No 23/2002.).

## Results

Four hundred ticks were collected from 95 birds, belonging to 11 species. Four species of ticks were found: *Ixodes ricinus*, *I. arboricola*, *I. redikorzevi*, *Haemaphysalis concinna* (Table 1). *Ixodes ricinus* was the most abundant tick, infesting 84.2 % (80/95, CI: 75.4–90.6) of wild birds, mainly as larvae and nymphs. Regarding host preferences, this species also showed the widest host range, parasitizing altogether ten bird species (data not shown).

**Table 1:** Number of *Rickettsia* spp. - infected ticks collected from birds in the Danube Delta, Romania (total number of analyzed ticks in parentheses)

	Developmental stage			
Tick species	Larva	Nymph	Female	Total
*Haemaphysalis concinna*	1 (2)	–	–	1 (2)
*Ixodes arboricola*	5 (10)	1 (15)	–	6 (25)
*Ixodes redikorzevi*	1 (4)	–	–	1 (4)
*Ixodes ricinus*	1 (181)	46 (180)	1 (8)	48 (369)
Total	8 (197)	47 (195)	1 (8)	56 (400)

All ticks were tested for the presence of SFG rickettsiae. Overall 14 % (56/400, CI: 11.7–29.1) of ticks were infected by *Rickettsia* spp., with significantly more nymphs hosting rickettsial infection compared to larvae (47 *vs* 8; *P* < 0.001). The prevalence of *Rickettsia* spp. infection in larvae was 4.0 % (8/197, CI: 1.7–7.3), in nymphs it was 24.1 % (47/195, CI: 18.9–31.2), while in adult females it was 12.5 % (1/8, CI: 0.6–5.0) (Table 1).

The prevalence of rickettsial infection was 14.4 % (48/369, CI: 11.1–18.4) for *I. ricinus*, and 24.0 % (6/25, CI: 7.2–40.7) for *I. arboricola*, while from each of the other two tick species, one infected individual was detected (Table 1). Positive samples were yielded by a proportion of 51.8 % (29/56) of ticks collected during spring and 48.2 % (27/56) collected during autumn. Significantly more ticks in nymphal stage were hosting *Rickettsia* spp. infection in spring, compared to autumn (*P* < 0.001), while no differences were found among the infection rate of the other development stages. A significantly higher proportion of birds was carrying *Rickettsia* spp.-infected ticks in spring, than in autumn (*P* < 0.001).

### DNA sequencing

The 56 positive samples were sequenced (Table 2) and nucleotide sequences were compared to those available in GenBank^TM^. BLAST analysis of these sequences showed 100 % similarity with *R. monacensis* (accession nos LN794217.1 and NR_115686.1), *R. helvetica* (accession nos KJ740388, KJ577822, L36212.1 and DQ910785), *R. slovaca* (accession no. L36224.1) and 99 % with *R. massiliae* (accession no. NR_074486), respectively. Only those samples, where similarity of 99 % or above was achieved (47/56) were assigned to a particular species [20]. Thus, the rickettsiae were identified as follows: *R. monacensis* in 29 ticks, *R. helvetica* in 13 ticks, *R. massiliae* in three ticks and *R. slovaca* in two ticks. No co-infection was detected in any of the sampled ticks. There were several cases where bird hosting multiple ticks, were carrying ticks with more than one rickettsial agent (e.g. individual ticks with one *Rickettsia* spp. each). We submitted the nucleotide sequences for all four *Rickettsia* species identified to GenBank™. The nucleotide sequence accession numbers are as follows: KR906075 for *R. helvetica*, KR906079 for *R. monacensis*, KR906080 for *R. slovaca* and KR906076 for *R. massiliae*.

**Table 2:** The distribution of different *Rickettsi*a spp. among ticks collected from birds in the Danube Delta, Romania (no co-infection was noted)

		Spotted Fever Rickettsiae			
Tick species	No. of ticks	*R. helvetica*	*R. massiliae*	*R. monacensis*	*R. slovaca*
*Haemaphysalis concinna*	2	–	–	1	–
*Ixodes arboricola*	25	4	1	1	–
*Ixodes redikorzevi*	4	1	–	–	–
*Ixodes ricinus*	369	8	2	27	2
Total	400	13	3	29	2

The seasonal distribution of different *Rickettsia* spp. was heterogeneous, with some species occurring both in spring and in autumn, while some occurred only in one season. Most of the *R. monacensis* - infected ticks were found in spring (75 % of occurrences), while more *R. helvetica*-infected nymphs were found in autumn (84 % of occurrences) than in spring. *Rickettsia slovaca* was found only in spring while *R. massiliae* was found only in autumn.

There was no statistically significant difference between the prevalence of *Rickettsia* spp. in ticks of migratory *vs* breeding bird species, although migratory birds were carrying ticks with more diverse *Rickettsia* species (4 *vs* 1 *Rickettsia* spp., with only *R. helvetica* found in ticks of breeding birds, Table 3). A total of six bird species was hosting ticks infected by rickettsial pathogens, with one species, the common blackbird (*Turdus merula*) carrying ticks infected with three species of *Rickettsia* spp. in spring migration and two species of *Rickettsia* spp. in autumn, while the song thrush (*Turdus philomelos*) was recorded to carry ticks with three *Rickettsia* spp. in autumn (Table 3). Generally, ticks hosted by birds with more than one tick tended to be infested by more *Rickettsia* species, with higher rickettsial prevalence associated to bird species carrying more ticks. However, we found no correlation between the prevalence of tick infestation and prevalence or number of *Rickettsial* species in ticks. In the meantime, the intensity of tick infestation (mean number of ticks on parasitized birds) was a good indicator of rickettsial species diversity of ticks hosted by individual bird species (Table 3, *P* < 0.01).

**Table 3:** Species and numbers of individuals of birds hosting ticks, with prevalences of SHG rickettsiae and details on life history status (migrant/breeding), migratory season (spring/autumn) and *Rickettsia* spp. harbored

	Tick presence			*Rickettsia* spp. presence			
Host species	No. of birds examined	Prevalence (%)	Intensity	Spring		Autumn	
				Prevalence (%)	*Rickettsia* species	Prevalence (%)	*Rickettsia* species
*Cyanistes caeruleus*	29	6.90	1				
*Emberiza schoeniclus*	11	9.10	1				
*Erithacus rubecula*	352	8.20	5.9	20.0	*monacensis*	3.1	*monacensis, helvetica*
*Ficedula hypoleuca*	10	10.00	2				
*Panurus biarmicus*	306	0.06	1			100	*helvetica*
*Parus major*	16	50	1.9			8.3	Unidentified *Rickettsia* spp.
*Phylloscopus collybita*	96	0.10	1			100	Unidentified *Rickettsia* spp.
*Regulus regulus*	47	4.20	1				
*Remiz pendulinus*	5	20.00	1				
*Turdus merula*	140	30.70	3.4	32.5	*monacensis, helvetica, slovaca*	14.2	*monacensis, helvetica*
*Turdus philomelos*	91	6.60	9.3	50.0	Unidentified *Rickettsia* spp.	16.9	*monacensis, helvetica, massiliae*

## Discussion

This is the first study in Romania showing that wild birds may contribute to the dispersion of *Rickettsia* spp. and that birds have an important role in the natural cycle of *Rickettsia* species associated with SFG rickettsioses in humans. Moreover, this is the first report and direct evidence of the presence of *R. massiliae* in Romania in ticks and also it represents the easternmost occurrence of this rickettsial agent in Europe [11]. SFG rickettsiae were already detected in more than 32 European countries, with most studies concentrating on questing ticks, while their occurrence in ticks of wildlife and especially their reservoirs are mostly unknown [11]. There are only a few studies which targeted ticks of migratory birds and their results suggested that birds may play an important role in the dispersion and maintenance of *Rickettsia* spp. in nature [7, 8, 21–24]. Moreover, studies suggest that birds may carry diverse tick communities and the associated rickettsial pathogen distribution is not uniform. In the present study, the majority of ticks was *I. ricinus* and represented 85.7 % (48/56) of all the infected ticks hosting four different rickettsial pathogens (*R. helvetica, R. massiliae*, *R. monacensis* and *R. slovaca*). Three different *Rickettsia* spp. were detected in *I. arboricola* ticks, with two of them unrecorded to date (*R. helvetica* and *R. massiliae*). While former studies regularly detected *Rickettsia* spp. in *I. arboricola* collected from migratory birds, these belonged to *R. monacensis* and ‘Candidatus *Rickettsia vini*’ [25–27]. *Ixodes redikorzevi* has a Palaearctic distribution, being primarily a small mammal parasite, with sporadic occurrences on humans [28, 29]. It is rarely recorded from birds [10] and the present study is the first to show its potential role in the maintenance and circulation of *R. helvetica* in nature. Although numerous studies report the presence of *H. concinna* in wild birds [7, 26, 30], with at least three different species of *Rickettsia* (*R. helvetica*, *R. heilongjiangensis* and *R. sibirica*) already identified in *H. concinna* ticks [7, 31, 32]; to the best of our knowledge this is the first detection of *R. monacensis* in this tick species.

*Rickettsia helvetica* was identified for the first time in 1979 in Switzerland from *I. ricinus*. Because transstadial and transovarial transmission of this rickettsia has been demonstrated in *I. ricinus*, this tick represents both a potential vector and a natural reservoir of *R. helvetica* [33]. This bacterium has already been detected in 24 European countries [11]. For approximately 20 years, *R. helvetica* was considered non-pathogenic; however, in 1999, it was found responsible for causing fatal perimyocarditis in several patients in Sweden [34], while recently it was found causing neuritis and Bell’s palsy [35]. *Rickettsia helvetica* was formerly found to be harbored by ticks collected from birds, with prevalence values ranging from 0.5 % in Germany [22] to 10.3 % in Russia [23], or as high as 51.4 % in Hungary [7]. In the Danube Delta, its prevalence was low in *I. ricinus* (1.9 %), comparable to Germany [22]. The seasonal distribution of *R. helvetica* in the current study is similar to the situation found [21] in Sweden, where autumn migrating passerines were carrying most ticks infected with this pathogen. The high prevalence of *R. helvetica* found in *I. arboricola* (16 %) may be an indication of reservoir competence for the song thrush. One individual belonging to this bird species harbored 32 ticks (27 *I. ricinus* nymphs and five *I. arboricola* larvae), of which one *I. ricinus* and four *I. arboricola* were infected by this pathogen. Even if the possibility of co-feeding transmission between the two tick species cannot be ruled out, this is however the first detection of *R. helvetica* in *I. arboricola*.

*Rickettsia monacensis* was detected for the first time in Germany in *I. ricinus*, it is a SFG *Rickettsia* which is distributed all over Europe [36]. This bacterium causes a zoonotic disease, characterized by acute fever (present in most cases), accompanied by chills, headache, photophobia, arthralgia, muscular pain, etc. [11]. The prevalence of *R. monacensis* in ticks varied between low (< 1 %, Mediterranean region, [8]) and very high (42.8 % in Germany, [22]). It is associated primarily with *I. ricinus* [36]. In the Danube Delta this pathogen was detected in three different tick species, with a prevalence of 2.7 % in *I. ricinus*. Similar prevalence was recorded in Russia [23] and Switzerland [24], while the seasonal distribution was similar (mostly in spring) on the Baltic Sea Island of Greifswalder Oie, in Germany [22]. Similarly, birds migrating through Ottenby, Sweden were carrying *R. monacensis* - infected ticks only in spring [21]. The common blackbird (*Turdus merula*) seems to be an important species for this pathogen, as most infected ticks (and also tick species) were found on this species of bird. *R. monacensis* was found in nine out of 14 nymphs harbored by an individual common blackbird, suggesting the reservoir competence for this species. This bird species also seem to be the most common bird-host for ticks carrying *R. monacensis* in most studies to date [7, 21, 24, 30].

*Rickettsia slovaca* was described for the first time in 1968, from *Dermacentor marginatus* in the former Czechoslovakia [37]. Human infections with this agent were recorded in France, Slovakia, Italy, Germany, Hungary, Spain and Poland [37]. *Rickettsia slovaca* has been found in *D. marginatus* and *D. reticulatus* ticks in Europe, with only these tick species being considered important vectors for this pathogen [38]. Its presence in ticks carried by birds was not recorded previously. This rickettsia is known to cause SENLAT syndrome in humans, with scalp eschar and neck lymphadenopathy commonly occurring in patients [33]. It was already recorded in Romania from free ticks [13], and recently was identified serologically from interned patients with symptoms of SENLAT syndrome in Bucharest [39].

*Rickettsia massiliae* was isolated from *Rhipicephalus sanguineus, Rh. turanicus, Rh. pusillus, Rh. bursa* and *I. ricinus* in eight European countries, including five islands: Sardinia and Sicily (Italy), the Canary Islands (Spain), Kephalonia (Greece), and Cyprus [11]. *Rickettsia massiliae*-infected ticks were collected from a wide range of animal hosts, including birds [6, 40]. It has a southern European and African distribution, with most records originating from the Mediterranean region in our continent [11]. Here we report its detection in two different tick species (*I. ricinus* and *I. arboricola*), with the easternmost occurrence on the European continent. Two occurrences were identified in ticks carried by two different song thrushes and the third one carried by a common blackbird, all cases recorded in autumn. *Rickettsia massiliae* causes a typical spotted fever disease (with febrile sensation, tick eschar with purpuric rash, asthenia, headache and, sometimes, face edema [33]. Although this rickettsial agent was not yet identified in Romanian ticks, its presence was recently recorded (and proved serologically) in six different patients interned with SENLAT syndrome [39].

In the current study, infections with several different tick-borne pathogens were determined for ticks carried by birds for the first time in Romania, highlighting the importance of migratory birds in the dispersion of SFG rickettsiae. Here we report the first individual records of different *Rickettsia* spp. in *H. concinna* (*R. monacensis*)*, I. arboricola* (*R. helvetica, R. massiliae*) and *I. redikorzevi* (*R. helvetica*), providing details also on their seasonal distribution. Birds migrating through the Danube Delta carried ticks with a number of four different rickettsial pathogens and migratory orientation and life history determined the rickettsial infection of ticks. Thus, *R. monacensis* and *R. slovaca* were the dominant pathogens in the pre-breeding period, when birds migrated from South and South-West towards North and North-East, while *R. helvetica* and *R. massiliae* were detected from birds in post-breeding migration, when birds travel to South and South-West. As migratory passerines usually have high migratory speed [41], they may easily transfer ticks to long distances, easily passing barriers otherwise insurmountable for ticks. By hosting ticks infected by diverse rickettsial agents, birds may be competent carriers of SFG rickettsiae over large distances or even between continents [21]. A number of studies proved the presence of multiple rickettsial pathogens in ticks of migratory birds [2, 6, 19–22, 24, 26], with several locations listed with more than one (up to three) *Rickettsia* spp. found [7, 8, 23, 32]. To the best of our knowledge the Danube Delta is unique in providing records of four different SFG rickettsiae identified in ticks collected from migratory birds. Although the prevalence rates found in this study were low, the high diversity and specific importance of different host species make the birds important as sentinels and also as long distance carriers for rickettsial pathogens.

## Conclusion

This study has shown that birds migrating through eastern Europe may carry ticks infected with a high diversity of rickettsial pathogens, with four *Rickettsia* spp. recorded. Migratory direction was important for pathogen burden, with seasonal differences in the occurrence of individual *Rickettsia* species. This is the first record of occurrence of R. *massiliae* in Romania, representing the easternmost record on the continent.
